# Exploring the effect of three scaffoldings on the collaborative problem-solving processes in China’s higher education

**DOI:** 10.1186/s41239-021-00273-y

**Published:** 2021-07-14

**Authors:** Fan Ouyang, Zixuan Chen, Mengting Cheng, Zifan Tang, Chien-Yuan Su

**Affiliations:** 1 College of Education, Zhejiang University, Hangzhou, 310058 Zhejiang China; 2 Department of Education, National University of Tainan, Tainan, Taiwan

**Keywords:** Collaborative problem-solving, Instructional scaffolding, Task-oriented scaffolding, Idea-oriented scaffolding, Online collaborative learning, Higher education

## Abstract

Collaborative problem-solving (CPS) engages students in solving ill-structured problems, creating group knowledge, and developing self-regulation and collaboration skills. Different scaffoldings, such as minimal-guided, task-oriented, and idea-oriented, can be used to facilitate students’ CPS activities, but their effects have not been comprehensively explored. In this research, we use minimally-guided, task-oriented, and idea-oriented scaffoldings to promote Chinese university students’ online CPS activities and use a multi-method approach to analyze the effects of three scaffolding on collaboration. The results indicate relatively complicated collaborative processes and outcomes supported by three scaffoldings. It is initially shown that the idea-centered scaffolding strengthens students’ connections between idea contribution, metacognitive regulation, and knowledge artifact behaviors, which are critical factors for improving the CPS quality. Based on the empirical research results, we conclude that future instructional design should carefully consider the educational culture, time constraint, and student regulation to better facilitate CPS practices.

## Introduction

Grounded upon the social perspective of learning (Vygotsky, 1978), computer-supported collaborative learning (CSCL) emphasizes small groups of people’s collaboration practices in coordinated activities to maintain mutual understandings, to advance joint meaning-making, and to create new knowledge and artifacts (Dillenbourg, 1999; Goodyear et al., 2014; Roschelle & Teasley, 1995). As one of the CSCL modes, collaborative problem-solving (CPS) engages people to work in groups to solve problems that are often open-ended and ill-structured, and beyond the existing skills and abilities of the individual learner (Brown et al., 1989; Kapur, 2008; Scardamalia & Bereiter, 2006). To facilitate a high quality of CPS, instructional scaffoldings, e.g., minimally guided instruction, task-oriented scaffolding, and idea-oriented scaffolding, are commonly used. However, previous empirical results varied about the effectiveness and efficiency of different instructional scaffoldings on collaborative learning practices (Hmelo-Silver & DeSimone, 2013; Kirschner et al., 2006; Stahl, 2009). From the research perspective, one of the reasons that result in the different conclusions owing to the complex, multi-dimensional characteristics of the collaborative learning activity, but most studies merely focus on one or two dimensions of the collaborative processes (Janssen et al., 2013). Filling the research and practice gaps, we designed a quasi-experiment research to provide groups with the minimally-guided, task-oriented, and idea-oriented scaffoldings during online CPS activities in China’s higher education. To better understand the effect of scaffoldings, we conducted a fine-grained, multidimensional analysis of the CPS process with a multi-method approach. Specifically, student groups’ collaborations were examined from the social, cognitive, metacognitive, behavioral, and temporal dimensions, supplemented with the analyses of groups’ collaborative products and students’ perceptions about collaboration. Based on the empirical research results, we provided pedagogical implications that help foster online collaborative problem-solving.

## Literature review

### Collaborative problem-solving processes

CSCL focuses on the collaborating groups’ meaning-making practices with the design and support of technological artifacts to mediate interactions and communications (Hmelo-Silver, 2004; Hmelo-Silver & DeSimone, 2013; Stahl, 2009). CPS, as one of the main CSCL modes, has been widely used in schools to improve student learning and performance (Kapur, 2008). CPS is defined as a group of learners building mutual understandings of a shared problem, pooling together their expertise, skills, and efforts, and come up with a final solution for the problem (Barron, 2000; Fiore et al., 2017; Hmelo-silver, 2004). CPS typically situates learning in solving real-world authentic, ill-structured problems, encourages students to create group knowledge, and develops responsibilities, self-regulation and collaboration skills for learning (Hmelo-Silver & DeSimone, 2013; Jonassen, 1997; Salomon, 1993). CPS has been widely used in K-12, higher education, and informal learning to improve students’ learning quality (e.g., Avry et al., 2020; Chang et al., 2017; Hong et al., 2011).

CPS is a complex, multidimensional, and multilevel practice that needs students’ coordination of the social, cognitive, metacognitive, and behavioral activities in a temporal fashion (Fiore et al., 2017; Hmelo-Silver & DeSimone, 2013; Stahl, 2009). First, CPS cannot be completed with a low level of social interaction and participation (Stahl, 2009). The group of students must participate in the social practices and interact with each other to jointly solve problems (Hakkarainen et al., 2013). Next, cognitive engagement to solve the ill-structured problems usually needs students’ exploration and understanding of the problem, proposition, and justification of the solutions, and the sustained development of new ideas and artifacts in the groups (Hakkarainen et al., 2013; Paavola et al., 2004; Scardamalia & Bereiter, 2006). Third, on the behavioral dimension, students need to take actions to externalize their knowledge in artifacts (e.g., concept map, writing document) and refer to peers’ behavior to coordinate their group activity and optimize the knowledge artifacts (Stahl, 2017). Moreover, to succeed in the group collaboration, students need to negotiate what to achieve as a group, plan, and implement problem-solving strategies, and monitor and reflect on the working progress (Järvelä & Hadwin, 2013; Winne et al., 2013). And the social, cognitive, behavioral, and metacognitive activities unfold in a time frame that enable students to coordinate their interactions, cognition, and actions to complete a high quality collaboration (Kapur, 2011; Lämsä et al., 2020).

### Effects of instructional scaffoldings

Different instructional strategies to scaffold the CPS processes have been implemented and studied. In general, scaffolding refers to a process in which an agent (e.g., the instructor, a peer and/or a computer system) helps a learner to complete the tasks that are challenging for the learner without any external assistance (Wood et al., 1976). In this research, the instructional scaffolding refers to the instructor’s procedural support in an online collaborative environment that guides students to engage in the CPS processes (see Hong & Lin, 2019). Because of the complex nature of CPS, although the minimally guided instruction is intuitively appealing for collaborative learning design, it may not always lead to desirable outcomes, such that instructors need to provide some forms of scaffoldings to support student collaboration (Hmelo-Silver & DeSimone, 2013; Hong & Lin, 2019; Kirschner et al., 2006). The task-oriented and the idea-oriented instructions are two primary approaches the instructors can use (Hong, 2011; Hong & Lin, 2019; Hong & Sullivan, 2009). In the task-oriented CPS activity, to achieve maximum efficiency of collaborative work, students are frequently asked to complete a project or solve a problem in the highly-structured group activities with some forms of group-working techniques, such as division of labor and scripted role-playing (Hmelo-Silver, 2004; Hong & Lin, 2019; Hong & Sullivan, 2009). For example, the task-oriented, role-assigned Jigsaw instruction has been widely used to engage students work in the expert groups and the jigsaw groups to explore, share and synthesize knowledge (Oshima et al., 2019). An alternative design of the CPS activity is based on the idea-centered collaboration that sees ideas as the core for students to create and build on, which emphasizes less-scripted, and more flexible, self-organized interactions in groups (Hong, 2011; Hong & Sullivan, 2009; Zhang et al., 2009). For example, the idea-centered knowledge building pedagogy engages students work in the knowledge forum to post their problems, produce initial ideas for problem-solving, and connect, revise, and synthesize ideas (Hong, 2011). In summary, while the instructional scaffoldings can take varying forms (e.g., scripts, prompts, tools), the task-oriented and idea-oriented scaffoldings are two primary means the instructors can use to design and organize the CPS activity.

However, previous empirical research indicates complicated effects of the task-oriented and idea-oriented scaffoldings on the collaborative learning processes and outcomes. For example, Hong (2011) compared the conventional task-based collaborative learning using the Jigsaw instruction and the idea-centered collaborative knowledge building; results showed that engaging students in idea-centered collaboration better enhanced their collaborative competencies, facilitated their peer interactions, and improved the idea improvement quality. However, some learning and instruction characteristics overlapped: the routines and procedures were still the unavoidable parts in idea-oriented learning, while knowledge advancement also occurred in task-oriented learning (Hong, 2011). Wang et al. (2017) examined the effect of student collaboration in the concept-oriented task (involving sharing information and knowledge) and design-oriented (involving task planning, monitoring and problem-solving) task; results showed that collaborative concept mapping functioned more effectively in the concept-oriented task than the design-oriented task, in terms of promoting students’ question-asking and positive motivations. But there were no significant differences in other social, cognitive, and emotional dimensions. Baghaei et al. (2007) designed an intelligent tutoring system to provide task-based and collaboration-based feedback messages to groups; empirical results showed that the use of the task-based structure achieved similar effectiveness on learning as the collaboration-based structure. Lin and Chan (2018) compared two key epistemic patterns during knowledge building, namely problem-centered uptake and theory‐building moves; their analysis showed that the higher‐quality discourse threads included more problem-centered uptake moves in which ideas were built more coherently on each other to address the central problem. Given the complexity, it is necessary to examine how different scaffoldings influence collaborative learning; in particular, a close examination of varied dimensions (i.e., social, cognitive, metacognitive, behavioral, and temporal) of CPS is critical for reaching a solid understanding.

### The analytical framework and methods of the CPS processes

The CSCL field has been promoting the use of multiple analytical methods to understand the complication of collaborative learning from the fine-grained, micro-level, and multi-dimensional perspectives (Borge & Mercier, 2019; Janssen et al., 2013; Suthers et al., 2013). More importantly, previous research in CSCL identifies primary dimensions of online collaborative learning processes (including social, cognitive, metacognitive, behavioral, and temporal) to assure students to effectively organize, coordinate, and contribute to the CSCL process in order to build group knowledge, solve collective problems, and achieve collective accomplishments (Garrison et al., 2000; Henri, 1992; Lämsä et al., 2020). Both qualitative and quantitative methods are used to reveal the interactions that can be attribute. On the one hand, the quantitative analysis approaches have been used to examine summative, sequential, temporal attributes of the group’s collaborative learning processes (Puntambekar, 2013). For example, the statistical analysis, sequential analysis, and social network analysis approaches have been used to investigate correlations between collaborative variables (Zemel et al., 2009), sequences of students’ knowledge contributions (Chen et al., 2017) and social interaction structures and participatory roles (Ouyang & Chang, 2019; Ouyang & Scharber, 2017), respectively. On the other hand, qualitative, ethnographic approaches (e.g., content or discourse analysis) have also been used to examine the micro-level turn-taking relevancies between the interactional, behavioral, and cognitive activities (Stahl, 2009; Zemel & Koschmann, 2013). Hong et al. (2011) collected teacher students’ online discussions, survey responses, and interviews to investigate their interaction patterns, reflective patterns, and knowledge building perceptions. Moreover, the analysis of the temporal dimension is critical to reveal collaborative processes that may be overshadowed through quantitative “coding-and-counting” approach (Chen et al., 2017; Kapur, 2011; Lämsä et al., 2020). Considering the multi-dimensional characteristics of CPS, merely focusing on one dimension of collaboration may cause inconclusive and incomprehensive results regarding the effects of instructional design; therefore, multiple analytical methods can complement each other in order to provide a more holistic, fine-grained analysis of the CPS process. Moreover, a better understanding of the effect of scaffoldings on the CPS process can improve the future pedagogical design, which is critical for facilitating a high-level quality of CPS.

To advance research, analysis, and practice of CPS, this quasi-experiment research used the minimally-guided, task-oriented, and idea-oriented scaffoldings to facilitate the CPS activity, and used a multi-method approach to examine the main dimensions of CPS practices under three scaffolding conditions. In the following sections, we introduced the research context, the analysis methods, procedures, and results and provide relevant implications.

## Methodology

### Research purpose and questions

To investigate the effect of different scaffoldings, we designed and facilitated collaborative problem-solving activities in China’s higher education context with the support of an online technological platform. Our research question was: *What were the differences of the effect of the minimally-guided, task-oriented, and idea-oriented scaffoldings on groups’ CPS processes?*

### Research context, participants and procedure

The research context was the online, synchronous CPS activity in China’s higher education context. The CPS activity belonged to a graduate-level course titled *Distance and Online Education*, offered in the 2020 Spring semester (8 weeks) by the Educational Technology (ET) program at a top research-intensive university in China. The course instructor (the first author) designed and facilitated the CPS activity to engage small groups (triads) to solve authentic problems instructors would face in distance and online education during COVID-19. The instructor designed eight ill-structured, open-ended problem cases, covering different subjects, student ages, and educational contexts. Cases included the design of online teaching components, (a) synchronous discussions, collaborative projects for mathematics, programming, engineering, geography classes, etc. (see “Appendix A” for an example). A research consent form was sent through the ET program’s social media (WeChat group) to invite students to participate in the research. Ten participants voluntarily participated and agreed with the data collections; one participant withdrew the participation in the middle of the semester, which was excluded from the research (see Table 1).

**Table 1:** Participant information

	Participant	Gender	Age	Status
Group A	A1	Female	32	Graduated student
	A2	Male	41	Part-time Master student
	A3	Male	25	Potential graduate student
Group B	B1	Female	24	Full-time Master student
	B2	Female	36	Full-time Ed.D. student
	B3	Male	23	Full-time Master student
Group C	C1	Female	24	Potential graduate student
	C2	Male	31	Full-time Ph.D. student
	C3	Female	27	Full-time Master student

**Fig. 1:**
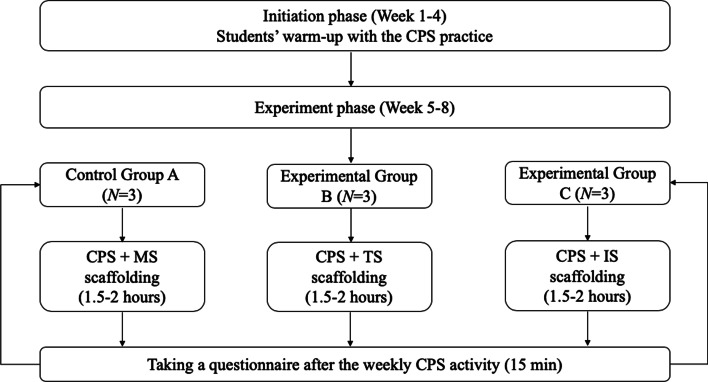
The research procedure

The primary instructional design of the 8-week CPS activities followed the problem-based learning cycle (Hmelo-silver, 2004): students first analyzed the problem scenario, then identified the critical aspects or knowledge needed to be addressed for the problem, next generated possible solutions through the concept mapping function, and finally reflected on the knowledge applied and/or created during the process. The problems were all ill-structured problems that did not have a fixed solution. The quasi-experiment design was structured into two phases (see Fig. 1). The first four weeks was designed as an initiation, warm-up phase for students to get familiar with the collaborative flow, online platform, and group work, because previous research indicated that Chinese students would be more used to learn with the highly-structured procedure rather than the open-ended, collaborative inquiry in small groups (Hong et al., 2011; Ouyang et al., 2020; Supanc et al., 2017). The instructor’s basic scaffoldings (e.g., providing relevant resources, explaining the problem-based learning procedures, solving technique issues, and reminding the remaining time) were provided to all groups in the initiation phase. The last four weeks were the experimental phase. There was one control group (i.e., Group A) with the minimal-guided scaffolding (MS), one experimental group (i.e., Group B) with the task-oriented scaffolding (TS), and another experimental group (i.e., Group C) with the idea-oriented scaffolding (IS) (see Fig. 1). Group A was the control group where students followed the regular CPS practice as they did in the initiation phase, supported with the instructor’s minimal guidance. Group B was offered with the task-oriented scaffolding, through which the instructor provided suggestions about critical aspects to be addressed and corresponding sub-tasks for students to complete. The scaffolding was offered in audio and text by the instructor every 10 min through the online platform. The prompts included *The overarching goal is* … *as a group*, *we plan to complete the task with step 1…, step 2…, and step 3*…, *Our strategy is* … *to complete the task 1/2/3*…, *Currently, we focus on the idea/question/solution of*… *What we have done well is* … *What we could improve is*… *Where we are now to achieve the goal*… *What we need to do next to complete the goal is…* Group C was offered with the idea-oriented scaffolding, through which the instructor asked students to identify their knowledge deficiency (i.e., the learning theories or instructional models that could underpin the problem-solving process) and explore and propose a theoretical or instructional framework to solve the problem. The scaffolding was offered in audio and text every 10 min through the online platform. The prompts included: *Is the current idea novel and interesting based on the framework? Can we improve the idea or framework in any way?*, *Is this idea workable?*, *Is this idea relevant?*, *Is this idea specific to the problem to be solved?* The crucial difference between those two scaffoldings is that the idea-oriented scaffolding used the idea-centered knowledge building pedagogy to empower students create new solutions based on the theoretical or instructional framework they chose (Hong & Sullivan, 2009), while the task-oriented scaffolding merely reminder students to complete the concept mapping activity through multiple steps rather than focusing on idea-centered knowledge building.

**Fig. 2:**
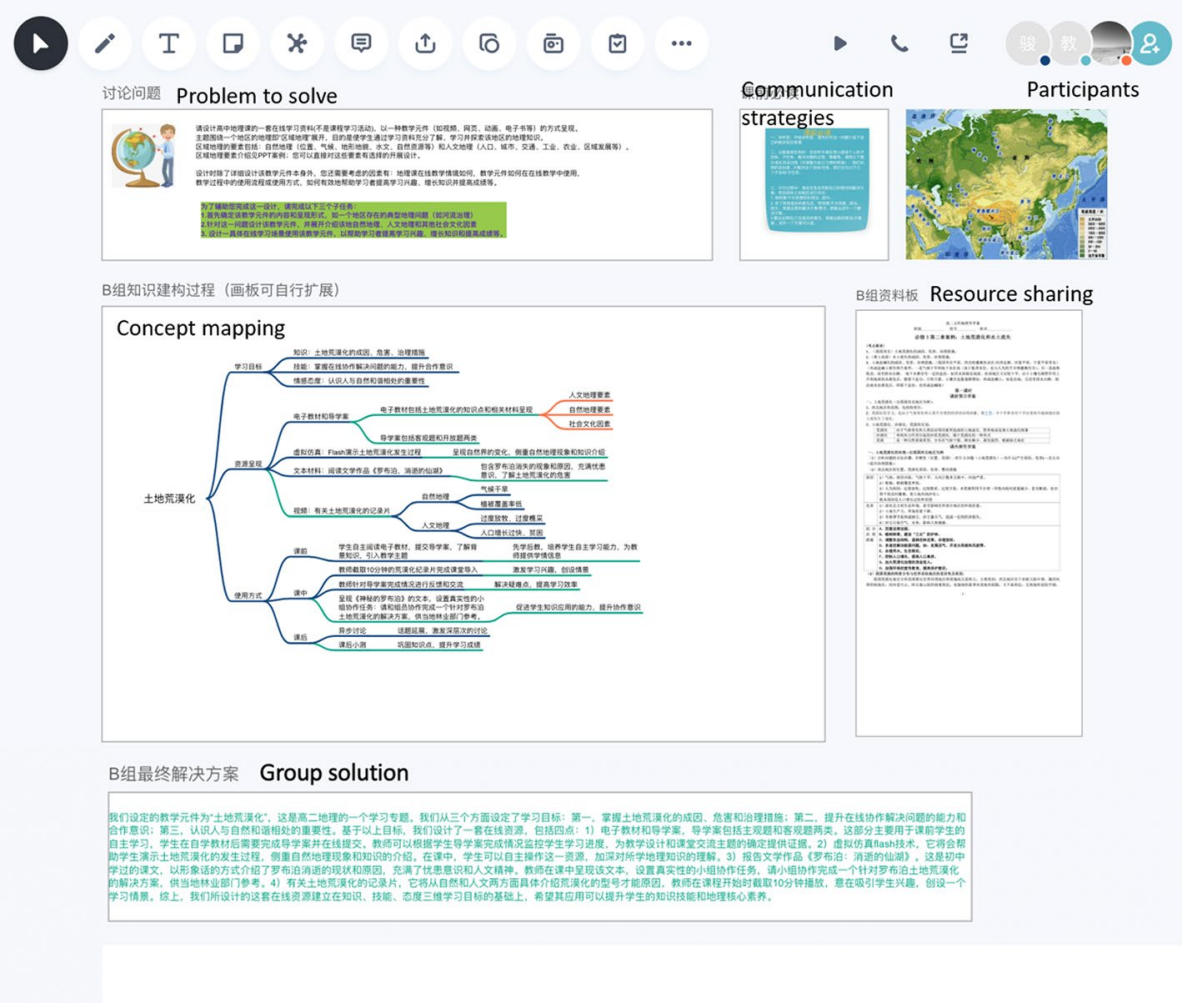
A group’s screenshot on the Huiyizhuo platform

The online platform *Huiyizhuo* (https://www.huiyizhuo.com/) was used to support the collaboration (see Fig. 2). *Huiyizhuo* provides text chatting, audio and video communication, concept map, note and comment, resource sharing, etc. In the CPS process, group members first communicated through audio and text chatting to determine how to proceed with the problem; then, groups shared resources, continued communications and constructed concept map to demonstrate their problem-solving processes; and finally wrote the groups’ solution proposal as a separated section on the platform. The concept map served as the main medium for participants to interpret the problem at hand, discuss and negotiate their understandings, present knowledge from multiple perspectives, identify misunderstandings and finally reach the group consensus of the solution (Engelmann & Hesse, 2010).

### Data collection and analysis

The data was collected in three ways. The original data was the computer screen recordings with audio (about 1.5 h/group/week), supplemented with the groups’ concept maps, solution write-ups, and students’ questionnaire responses after each week’s CPS activity (see “Appendix B”). The original data (i.e., computer screen recordings with audio) was transcribed to 12 excel files (4 files for each group) that recorded students’ online discourses and behaviors.

A multi-method approach was used to examine details of the groups’ collaboration from the social, cognitive, metacognitive and behavioral dimensions (see Table 2). First, social network analysis (SNA) was used to analyze the *social* characteristics that represented group interactions through oral communications and knowledge artifacts. The original data were transformed into a directed, weighted student–student network dataset. In the networks, the direction represented who responded to whom and built on whose work (i.e., bi-directional); tie weight represented the frequency of responses, replies and build-on work a participant makes to others (i.e., interaction frequency). We chose three network-level metrics to represent social characteristics, including the total interaction frequency, the average degree (calculated by outdegree and indegree with the Opsahl’s alpha of 0.5), the coefficient of variation (CV) of interaction (the standard deviation of student interaction frequency divided by its mean) (Opsahl, 2009; Ouyang, 2021; Ouyang & Scharber, 2017). The total interaction frequency represents the group’s overall social interaction levels; average degree represents the average level of interaction frequency in a group; CV represents the unbalanced attributes of interactions between students in a group (e.g., a larger CV indicates a more unbalance interaction in a group).

**Table 2:** The overall analytical framework

Data type	Data	Analytical methods	Analytical purposes
Process data	Computer screen recording data (with audio)	Social network analysis (SNA)	To analyze peer interactions through oral communications and constructions of concept map artifacts
	Audio data	Content analysis (CA)	To analyze the cognitive and metacognitive dimensions of the discourses
	Computer screen recording data	Clickstream analysis (CSA)	To analyze the behavioral dimension of students’ online platform behaviors
		Lag sequential analysis (LSA)	To analyze transitions of cognitive, metacognitive, and behavioral dimensions
Performance data	concept maps and solution write-ups	Product analysis (PA)	To analyze the final group products of concept maps and write-ups based on previous validated assessment standards
Self-reported data	Post-course questionnaire	Statistical analysis (SA)	To analyze student perceptions about group collaborative quality, student engagement level, and usefulness of the scaffolding

Then, content analysis (CA) was used to examine the *cognitive* and *metacognitive* characteristics of three groups (see Table 2). Referring to a previous study (Ouyang & Chang, 2019), we used the CA approach to analyze the *cognitive* dimension that represented students’ knowledge contributions in the superficial, medium, and deep levels (see Table 3). Referring to the previous research (Malmberg et al., 2017), we used the CA approach again to analyze the *metacognitive* dimension that represented students’ regulation of their collaborations. Three codes of the *metacognitive* dimension were task understanding, goal setting and planning, and monitoring and reflection (see Table 3). In the CA process, the unit of analysis was the *sentence* (i.e., a full sentence spoken by a participant). One sentence could be assigned to more than one code if the cognitive or metacognitive contributions occurred during student discourses. Next, we used the clickstream analysis (CSA) to analyze the *behavioral* dimension, including resource management, concept mapping and observation. The *unit of analysis* of the behavior was a participant’s mouse clicking or moving operation on the platform. We kept the *cognitive*, *metacognitive* and *behavioral* codes for each group in the excel files according to the time framework.

**Table 3:** The analysis dimensions and descriptions

Dimension	Code	Description	Examples
Cognitive	Superficial (KS)	A participant simply shares information, presents (dis)agreement, asks questions, or seeks for clarifications, without explicit statement of his/her own ideas, arguments, or perspectives	What does it mean…? Yes, I agree with you
	Medium (KM)	A participant elaborates his/her own ideas, arguments, or perspectives without detailed explanations, supports of resources, statistics or personal experiences	I feel like… it's just a different learning activity
	Deep (KD)	A participant explicitly elaborates his/her own ideas, arguments, or perspectives with detailed explanations, supports of resources, statistics or personal experiences	I think students need to apply those knowledge in their lives in order to understand them…because the application process can help them make sense of…
Metacognitive	Task understanding (TU)	A participant activates previous knowledge of the task and contents, thinks about the purpose of the task, identifies what should be done for this task, reads and interprets the questions or instructions	We need to define the concept of the topic first…
	Goal setting and planning (GSP)	A participant thinks about what documents and resources are needed, plans or divides the task, plans and discusses what to do next	We're going to break it down into three dimensions…first, we need to…
	Monitoring and reflection (MR)	A participant monitors and evaluates the progress toward the criteria set for the task, evaluates the time schedule set for finishing the task; summarizes what has been done and what needs to be done	Regarding this question, I think what we have done is still the same thing, we need to break out of that mindset
Behavioral	Resource management (RM)	A student searched, shared or read resources on the platform or through the Internet	I find an article about our topic This academic article is about……
	Concept mapping (CM)	A participant created, modified, or commented on the concept map	Creating a concept map through huiyizhuo functions
	Observation (OB)	A participant moved the mouse over the platform to observe without any operations	Moving the mouse over the huiyizhuo platform without speaking

It is worth mentioning the analysis process was iterative. The first author coded Week 5’s transcribed data first and proposed an initial coding scheme. Then, other four authors re-recorded Week 5’s data in terms of the initial coding scheme, had multiple meetings to solve discrepancies, and came up with the final coding scheme (see Table 3). Krippendorff’s (2004) alpha reliability was 0.735 among four authors at this phase’s analysis. Finally, all authors coded the whole dataset separately, cross-checked the analysis results, and consulted with the first author to solve discrepancies.

Finally, lag-sequential analysis (LSA) is used to examine the sequential contingencies of cognitive, metacognitive and behavioral events, including the direct (lag = 1) and indirect (lag = 2) sequential transitions between codes (Chen et al., 2017). We focused on the transitions between three different dimensions (i.e., cognitive, metacognitive, and behavioral) and between three different codes under each dimension (see Table 3). There were 9 possible transition patterns among three dimensions, as well as 9 possible transition patterns under each dimension. By checking the total dataset of 12 excel files, we guaranteed that a sufficient data volume included by checking that the total dataset was at least 10 times the number of transition cells (Lämsä et al., 2020). Here, we used the Yule’Q to calculate the strength a code transitioned to another code. Yule’s Q represents the strength of transitional association because it controls for base numbers of contributions and is descriptively useful (with a range from − 1 to + 1 and 0 indicating no association). To detect the differences of groups’ patterns, we specifically examined three types of sequential transitions, including the transitions between three dimensions, between three codes under each dimension, and nine codes across three dimensions.

Complementary to the collaborative pattern analysis, we evaluated groups’ collaborative performances and perceptions. The evaluations of concept map artifacts as the primary, complemented with the solution write-ups, were conducted as the group performances. Adapting a previous assessment approach (Novak & Cañas, 2008), we used product analysis (PA) to assess the concept map in terms of three dimensions, i.e., propositions, hierarchy, and examples, and used the cognitive dimension in the coding scheme (see Table 3) to analyze the groups’ write-up scores. The unit of analysis was a sentence separated by a semicolon or a period in the write-up that represented a complete idea. Two authors scored the concept maps and write-ups independently and reached an agreement if there were differences. Next, we analyzed students’ questionnaire responses to demonstrate student perceptions about group collaborative quality, their engagement level, and the usefulness of the scaffoldings.

## Results

**Fig. 3:**
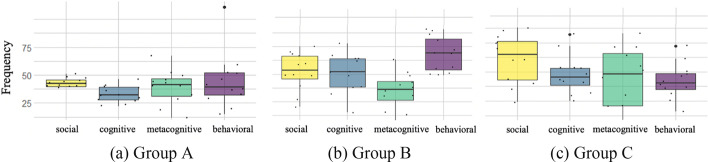
Three groups’ distribution boxplots of social, cognitive, metacognitive and behavioral frequencies. Each box represents students’ code frequencies (minimum, median, and maximum values) throughout four weeks on one dimension

An overview of the comparison of three groups’ *social*, *cognitive*, *metacognitive*, and *behavioral* contributions during four weekly CPS is presented in the distribution boxplots (see Fig. 3). Group A had the lowest level of social and cognitive contribution; Group B had the highest level of cognitive and behavioral contribution and the lowest level of metacognitive contribution; Group C had the highest social and metacognitive contribution and the lowest level of behavioral contribution. The overall results indicate rather complicated collaborative results of three groups. We make further examinations of sequential transitions of each dimension and across dimensions as followings to better understand the collaborative patterns.

### Social dimension

**Fig. 4:**
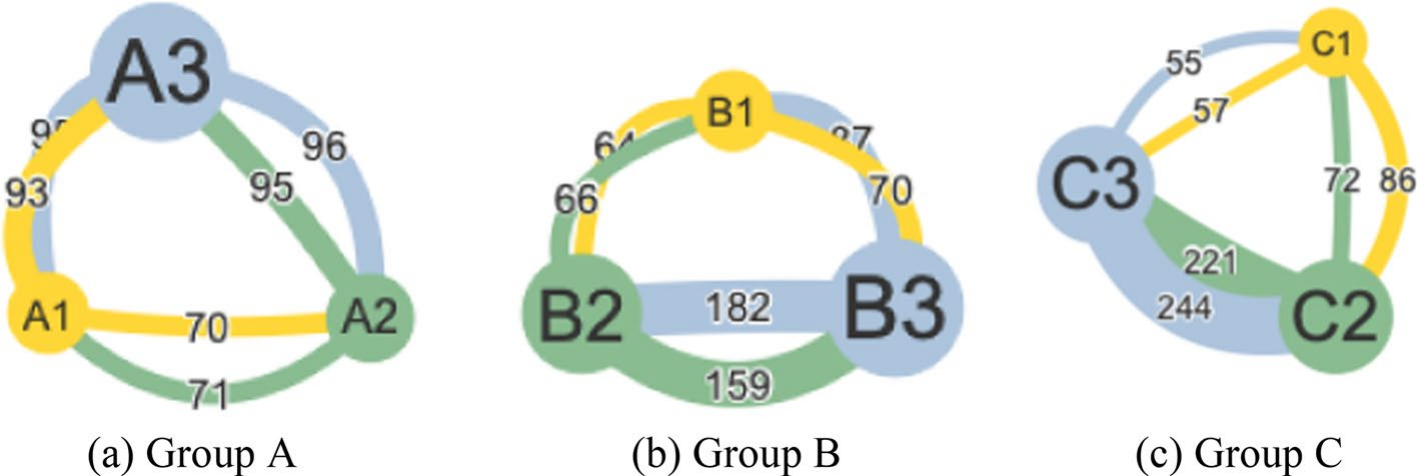
Social interaction network of three groups. The node size represented interaction frequencies; the number on the tie represented the interaction a student had to another student; the direction of a tie should be read from the node with the same color of the tie to the node with a different color (e.g., C3 sent 244 replies to C2)

First, on the social dimension, Group C was the most active group throughout the four weekly CPS activities, reflected by the highest frequency of student interactions (*freq.* = 735, *M* = 183.75, *SD* = 26.85), followed by Group B (*freq.* = 628, *M* = 157.00, *SD* = 23.04) and Group A (*freq.* = 520, *M* = 130.00, *SD* = 5.16) (see Fig. 4). Group C had the highest level of average degree (*outdegree* = 21.9, *indegree* = 21.7), followed by Group B (*outdegree* = 20.3, *indegree* = 20.4) and Group A (*outdegree* = 18.6, *indegree* = 18.6). Group C also had the highest CV value (*CV* = 1.42), indicating a high level of dispersion of students’ interaction frequencies, followed by Group B (*CV* = 0.50) and Group A (*CV* = 0.15). Overall, Group C was the most interactive group among three groups, followed by Group B and Group A (see Fig. 4).

### Cognitive dimension

On the cognitive dimension, Group B had the highest frequency of cognitive contribution throughout four weekly CPS activities (*freq*. = 610, *M* = 152.50, *SD* = 32.28), closely followed by Group C (*freq*. = 575, *M* = 143.75, *SD* = 31.62), and Group A (*freq*. = 397, *M* = 99.25, *SD* = 17.84). The sequential analysis results showed that all three groups had the direct transitions on the cognitive dimension (i.e., *Cog-* > *Cog*), where Group C had the strongest value (*M* = 0.61, *SD* = 0.10), followed by Group A (*M* = 0.53, *SD* = 0.18) and Group B (*M* = 0.26, *SD* = 0.23) (see Table 4). Group C also consistently had the strong transition on cognitive dimension, reflected by the small SD value. In addition, results of ANOVAs and ANCOVAs showed a significant difference among three groups on the *Cog-* > *Cog* transition (*p* < 0.05), where Group C had the highest value, regardless of taking *time* as covariate or not.

**Table 4:** Direct transitions of the cognitive, metacognitive and behavioral dimensions (Group A, Group B, Group C)

Current move	Upcoming move		
	Cog	Meta-Cog	Beh
Cog	0.53 (0.18), 0.26 (0.23), 0.61 (0.10)	− 0.25 (0.32), 0.14 (0.51), − 0.45 (0.09)	− 0.30 (0.25), − 0.34 (0.21), − 0.31 (0.10)
Meta-Cog	− 0.40 (0.22), − 0.05 (0.16), − 0.39 (0.18)	0.36 (0.33), 0.14 (0.46), 0.46 (0.13)	− 0.07 (0.26), − 0.14 (0.32), − 0.10 (0.12)
Beh	− 0.17 (0.16), − 0.21 (0.13), − 0.38 (0.03)	− 0.18 (0.17), − 0.36 (0.13), − 0.04 (0.12)	0.30 (0.17), 0.40 (0.15), 0.40 (0.03)

The values are shown as Mean (SD)

We further analyzed the direct transitional patterns between *KS*, *KM*, and *KD* under the cognitive dimension. All three groups had the direct transitions from *KS* to *KS* and *KM* to *KM*, but no transition or low-level *KD-* > *KD* transition (see Table 5). Among three groups, Group C had the strongest *KS-* > *KS* transition (*M* = 0.48, *SD* = 0.22) and *KM-* > *KM* transition (*M* = 0.39, *SD* = 0.32). Group A and Group B had no direct *KM-* > *KD* transition; in contrast, Group C had the strongest *KM-* > *KD* transition (*M* = 0.52, *SD* = 0.62). Results of ANOVAs and ANCOVAs (taking *time* as the covariate) indicated significant differences among three groups on the *KD-* > *KD* transition (*F* = 4.87, *p* < 0.05). Further examinations showed a significant difference between Group A and Group B, regardless of taking *time* as the covariate or not (*p* < 0.05) (see Table 5).

**Table 5:** Direct transitional patterns of the cognitive dimension (Group A, Group B, Group C)

Current move	Upcoming move		
	KS	KM	KD
KS	0.36 (0.13), 0.23 (0.29), 0.48 (0.22)	− 0.35 (0.24), − 0.22 (0.27), − 0.46 (0.24)	0.03 (0.70), 0.07 (0.31), − 0.46 (0.68)
KM	− 0.34 (0.13), − 0.26 (0.17), − 0.43 (0.28)	0.35 (0.19), 0.24 (0.14), 0.39 (0.32)	− 0.07 (0.58), − 0.12 (0.14), 0.52 (0.62)
KD	− 0.13 (0.27), 0.11 (0.22), − 0.06 (0.92)	0.11 (0.19), − 0.13 (0.16), 0.10 (0.92)	− 0.61 (0.79), 0.06 (0.30), − 0.01 (0.30)

The values are shown as Mean (SD)

### Metacognitive dimension

On the metacognitive dimension, Group C had highest metacognitive contributions (*freq*. = 523,* M* = 130.75, *SD* = 25.36), followed by Group A (*freq*. = 473, *M* = 118.25, *SD* = 35.34) and Group B (*freq*. = 411, *M* = 102.75, *SD* = 29.58). The sequential analysis results showed that all three groups had the direct metacognitive transitions (see Table 4). Among three groups, Group C had the strongest metacognitive transition (*M* = 0.46, *SD* = 0.13), followed by Group A (*M* = 0.36, *SD* = 0.33) and Group B (*M* = 0.14, *SD* = 0.46) (see Table 4). Group C also consistently had the strong transition, reflected by the small SD value. Multiple ANOVAs and ANCOVAs indicated no significant differences between three groups, regardless of taking *time* as the covariate or not.

We further analyzed the direct transitional patterns between *TU, GSP,* and *MR*. All three groups had the direct transitions from *TU* to *TU*, *GSP* to *GSP*, and *MR* to *MR* (see Table 6). Group A had the highest *GSP-* > *GSP* transition (*M* = 0.68, *SD* = 0.12) and the highest *MR-* > *MR* transition (*M* = 0.72, *SD* = 0.10); while Group B had the highest *TU-* > *TU* transition (*M* = 0.86, *SD* = 0.12). There were no direct transitions between two different codes on the *metacognitive* dimension. The multiple ANOVA and ANCOVA analyses (taking *time* as the covariate) found no significant differences among the three groups on any metacognitive dimension transitions.

**Table 6:** Direct transitional patterns of the metacognitive dimension (Group A, Group B, Group C)

Current move	Upcoming move		
	TU	GSP	MR
TU	0.79 (0.13), 0.86 (0.12), 0.42 (0.95)	− 0.39 (0.32), − 0.28 (0.44), − 0.06 (0.73)	− 0.39 (0.29), − 0.64 (0.56), − 0.74 (0.30)
GSP	− 0.40 (0.26), − 0.35 (0.27), − 0.14 (0.76)	0.68 (0.12), 0.39 (0.24), 0.54 (0.08)	− 0.54 (0.22), − 0.28 (0.18), − 0.44 (0.15)
MR	− 0.55 (0.37), − 0.83 (0.33), − 0.74 (0.30)	− 0.59 (0.12), − 0.19 (0.28), − 0.47 (0.17)	0.72 (0.10), 0.43 (0.35), 0.62 (0.08)

The values are shown as Mean (SD)

### Behavioral dimension

On the behavioral dimension, Group B had the highest frequency (*freq.* = 818, *M* = 204.50, *SD* = 19.46), followed by Group A (*freq*. = 533, *M* = 133.25, *SD* = 47.93) and Group C (*freq*. = 503, *M* = 125.75, *SD* = 37.08). The sequential analysis results showed that all three groups had the direct behavioral transitions (i.e., *Beh-* > *Beh*), where Group C had the strongest *Beh-* > *Beh* transition (*M* = 0.40, *SD* = 0.03), followed by Group B (*M* = 0.40, *SD* = 0.15), and Group A (*M* = 0.30, *SD* = 0.17) (see Table 4). Group C also consistently had the strong transition, reflected by the small SD value. In addition, multiple ANOVAs and ANCOVAs indicated no significant differences among three groups.

We further analyzed the direct transitional patterns between *RM, CM,* and *OB*. Multiple ANOVA analyses showed that there were significant differences between three groups on *OB-* > *CM* transition (*F* = 6.89, *p* = 0.02), where Group C had the strongest *OB-* > *CM* transition (*M* = 0.20, *SD* = 0.24). Further examination indicated significant differences of *OB-* > *CM* transition between Group A and Group B (*p* < 0.05) and between Group A and Group C (*p* < 0.05), regardless of taking *time* as the covariate or not (see Table 7).

**Table 7:** Direct transitional patterns of the behavioral dimension (Group A, Group B, Group C)

Current move	Upcoming move		
	RM	CM	OB
RM	− 0.54 (0.93), 0.85 (0.10), 0.30 (0.89)	− 0.74 (0.37), − 0.29 (0.24), − 0.37 (0.50)	− 0.38 (0.84), − 0.60 (0.27), − 0.77(0.32)
CM	− 0.62 (0.44), − 0.74 (0.33), − 0.71 (0.43)	0.47 (0.27), 0.09 (0.38), − 0.13 (0.38)	− 0.49 (0.23), 0.19 (0.34), 0.30 (0.20)
OB	− 0.49 (0.63), − 0.52 (0.28), − 0.63 (0.43)	− 0.44 (0.30), 0.06 (0.23), 0.20 (0.24)	0.45 (0.29), 0.30 (0.22), − 0.12 (0.24)

The values are shown as Mean (SD)

**Fig. 5:**
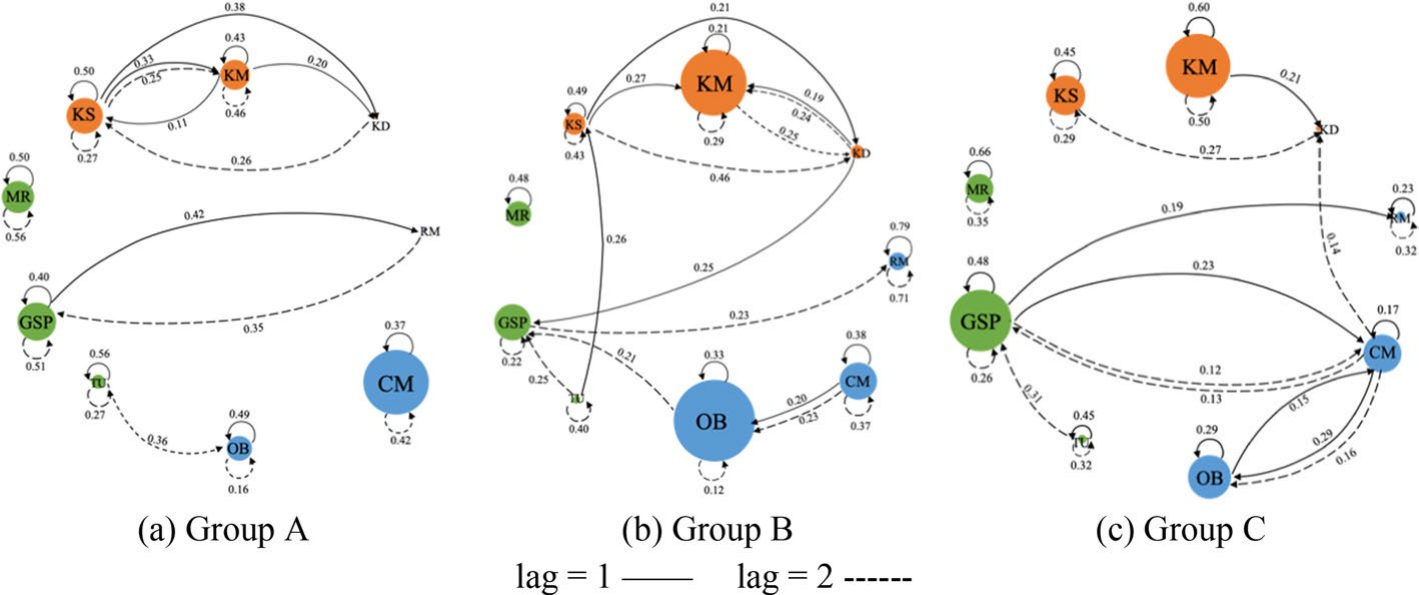
Direct and indirect sequential transitions across nine codes. The node size represents the average code frequency of the groups throughout four CPS activities. The values on the solid and dashed lines represent Yule’s Q values when lag is set to 1 and 2

We further examined the sequential transitions between 9 codes across three dimensions (see Fig. 5). Group A had 12 types of direct sequential transitions (lag = 1), with the strongest transition of *TU-* > *TU* (*Yule’Q* = 0.56), followed by *KS-* > *KS* (*Yule’Q* = 0.50) and *MR-* > *MR* (*Yule’Q* = 0.50); Group A had 9 types of indirect sequential transitions (lag = 2), with the strongest transition of *MR-* > *MR* (*Yule’Q* = 0.56), followed by *GSP-* > *GSP* (*Yule’Q* = 0.51), and *KM-* > *KM* (*Yule’Q* = 0.46). Group B had 12 types of direct sequential transitions (lag = 1), with the strongest transition of *RM-* > *RM* (*Yule’Q* = 0.79), *KS-* > *KS* (*Yule’Q* = 0.49) and *MR-* > *MR* (*Yule’Q* = 0.48); Group B had 14 types of indirect sequential transitions (lag = 2), with the strongest transition of *RM-* > *RM* (*Yule’Q* = 0.71), *KS-* > *KD* (*Yule’Q* = 0.46) and *TU-* > *TU* (*Yule’Q* = 0.40). Group C had 13 types of direct sequential transitions (lag = 1), with the strongest transition of *MR-* > *MR* (*Yule’Q* = 0.66), *KM-* > *KM* (*Yule’Q* = 0.60) and *GSP-* > *GSP* (*Yule’Q* = 0.48); Group C had 12 types of indirect sequential transitions (lag = 2), with the strongest transition of *KM-* > *KM* (*Yule’Q* = 0.50), *MR-* > *MR* (*Yule’Q* = 0.35), *RM-* > *RM* (*Yule’Q* = 0.32) and *TU-* > *TU* (*Yule’Q* = 0.32).

### Group performances and perceptions

Group C had the best collaborative performance among three groups. Group C (*M* = 89.75, *SD* = 34.95) ranked first among three groups of the concept map performance, followed by Group A (*M* = 88.50, *SD* = 24.15) and Group B (*M* = 65.00, *SD* = 20.15). Regarding the solution write-ups, Group C (*M* = 15.00, *SD* = 1.83) also ranked first, closely followed by Group B (*M* = 14.75, *SD* = 2.50) and Group A (*M* = 11.50, *SD* = 2.08). In addition, ANOVA results indicated that the scaffoldings had no statistical significances on students’ perceptions about the group’s collaborative quality on nine dimensions (see “Appendix B” part 1), where Group C (*M* = 8.46, *SD* = 1.03) reported the highest score, closely followed with Group B (*M* = 8.38, *SD* = 0.97) and Group A (*M* = 7.97, *SD* = 1.24). Group C’s students had the highest level of self-reported engagement score (*M* = 8.58, *SD* = 0.67), followed by Group B (*M* = 8.25, *SD* = 1.22), and Group A (*M* = 8.00, *SD* = 1.21). In an open-ended question about knowledge worker role, students in Group C perceived themselves as the *knowledge builder* in most cases. For example, C3 reported “…*most of the time, I think I was a knowledge builder… I integrated and synthesized others’ ideas… and finally we built knowledge together as a group*.”. Students in Group B perceived themselves as the *knowledge builder* in half of cases and the *knowledge user* in the other half. For example, B1 reported “*in some cases, I use the knowledge my partners provided to build the concept map… occasionally, I reflected on what I have learned previously and build new knowledge through communicating with my partners*”. Students in Group A in most cases perceived themselves as *knowledge user* and seldom perceived themselves as *knowledge builder.* For example, A1 responded “*I think most of the time I merely use the knowledge or information I already have… directly to the concept map*”. Finally, no statistical significances were found among the three groups about the usefulness of the scaffolding. Group B (*M* = 8.25, *SD* = 0.62) held the most positive attitude towards the usefulness of the scaffolding, followed by Group C (*M* = 7.67, *SD* = 1.56), and Group A (*M* = 7.35, *SD* = 0.88).

## Discussions and implications

This quasi-experiment research designs collaborative problem-solving activities supported with the minimally-guided, task-oriented, and idea-oriented scaffoldings in an online platform, and uses a mixed-method to examine the effect of three scaffoldings on the CPS processes, performances and perceptions. Consistent with previous research (e.g., Hong, 2011; Wang et al., 2017; Zhang et al., 2009), the research results indicated that the idea-centered collaboration had the best effect on the CPS process. Group C, with the idea-oriented scaffolding, formed the most dense student–student interaction network, through which they intensively built knowledge together (reflected by the strong cognitive transition and transitions between KS, KM and KD codes), made group regulation for the knowledge building process (reflected by the highest metacognitive contributions and the strongest metacognitive transition), and intensively took actions to work collaboratively through concept mapping (reflected by the strongest behavioral transition and the diverse behavioral transitions involving CM) (see Fig. 5). Group C also achieved the highest level of collaborative performance, perceived the best group collaborative quality, and self-reported the highest level of engagement.

Second, the task-oriented scaffolding results in a relatively complicated collaborative outcome. Group B, with the task-oriented scaffolding, formed the second interactive network, through which they had a high yet discrete cognitive pattern (reflected by the highest level of the overall cognitive contribution but the lowest level of transition between cognitive codes), a low and discrete regulation for the problem-solving process (reflected by the lowest metacognitive contribution and transition), but actively took actions to solve problems on the platform (reflected by the highest behavioral frequency). Moreover, Group B had the lowest score of the concept map but the highest score of the solution write-up, which implied that Group B’s students tended to merely focus on completing the tasks rather than exploring ideas and solutions on the concept map. Consistent with previous research, the result indicated that too much guidance would help students to stay on track of the task, but lessen the quality of open-ended inquiry, e.g., knowledge building (Dillenbourg, 2002).

Third, consistent with previous research, the result shows that CPS without additional instructional supports is less likely to lead to desirable outcomes (Kirschner et al., 2006). Group A, with the minimal-guided scaffolding, formed the least interactive network, through which students did not actively build knowledge to solve problems compared to the other two groups (reflected by the low-level cognitive contribution), had the middle level of group regulation (reflected by metacognitive contribution and transition), as well as the middle-level behavioral contribution and transition. The nine-code transition results showed that Group A had the least diverse behavioral transitions compared to the other two groups; notably, there were no behaviors connected to concept mapping (see Fig. 5). However, Group A achieved a good collaborative outcome regarding the concept mapping (ranked second among three groups). Overall, we conclude that the effect of three scaffoldings on the CPS process and quality is rather complicated.

In addition, student perceptions show that although the idea-oriented group has the best collaborative process and outcome, they do not perceive the idea-oriented scaffolding as useful as the task-oriented scaffolding. In contrast, although the task-oriented scaffolding may not be beneficial for achieving a high quality of problem-solving, students are more prone to employ the highly-structured, task-oriented, and goal-focused learning procedure in collaboration (Hong et al., 2011; Supanc et al., 2017). One explanation of this phenomenon is centered on China’s educational culture: when group collaboration is applied, it aims to complete clearly-defined tasks, solve well-structured problems, or achieve mastery of specific textbook knowledge with an efficiency that cannot be achieved by individuals (Hong, 2011). The idea-centered epistemological perspective is new to students because it sees knowledge as tentative, improvable and subject to change (Hong & Lin, 2019), such that student groups may experience difficulties in transforming the idea-centered epistemology to the actual collaborative process, particularly for students who are less experienced of this pedagogy (Avry et al., 2020; Ouyang & Chang, 2019; Rummel & Spada, 2005). In addition, the idea-oriented collaborative learning needs to go beyond fixed learning schedules to facilitate an emergent, self-organizing, and opportunistic way of working collectively, which is not easy for students to achieve in the short term (Hong & Sullivan, 2009; Zhang et al., 2009). Taking Group B, for example, Group B has a certain level of indirect transition in the cognitive dimension (between KM and KD) (see Fig. 5), and a low level of metacognitive regulation. This result indicates that students may need more time and better self-regulation skills to transform into the idea-centered form of collaborative learning. Pedagogical implications provided below can be beneficial for fostering CPS with the support of instructional scaffoldings.

Overall, the results indicate that the instructor should use idea-centered scaffolding to promote positive effects on the collaborative learning performance, process, and quality. For students who are prone to structured instructions, the task-oriented scaffolding can be used and modified by integrating idea-centered elements in order to help students achieve a better knowledge inquiry and construction. In addition, the instructor should make better preparations and take more time to situate students in the idea-centered knowledge building pedagogy (Lin & Reigeluth, 2016), particularly for students who are new to this pedagogy. The instructor should also encourage students to make shared references (Barron, 2000) and joint attentions (Çakır et al., 2009; Stahl, 2009) of knowledge building artifacts (e.g., the concept map), to develop self-regulation and group regulation skills (Winne et al., 2013), and to build collective responsibility during the CPS process (e.g., shared roles of knowledge builder) (Zhang et al., 2011). The educational culture, time constraint, and student regulation should be taken into consideration during CPS design and practice. The instructor should keep in mind that the collaborative mode of learning and instruction is not about giving students complete freedom; instead, the core philosophy is that student responsibility and agency of understanding, meaning-making, and knowledge building should be put at the center of educational practices (Bandura, 2001).

## Conclusions, limitations, and future directions

The recent calls for educational reform have highlighted the importance of transforming student learning from knowledge acquisition to participation and creation where students not only participate in social, collaborative group activities but also build and advance group knowledge during the process (Paavola et al., 2004). CPS is a commonly used learning mode to make this educational transformation, and varied scaffoldings are used to support CPS in order to improve students’ knowledge building and problem-solving. In this research, we use a mixed-method to examine groups’ CPS processes and initially find that the idea-centered collaboration strengthens the connections between idea contribution, metacognitive regulation and behaviors related to knowledge artifact, which is beneficial for improving the CPS quality. This research contributes to a more comprehensive understanding of the affordances and limitations of different scaffoldings on advancing collaborative learning. Although the results verify that the idea-centered scaffolding is the most useful strategy, this research was conducted in a small sample size of student groups during a relatively short time period. Future research should extend idea-centered collaboration to a longer-term and also expand the sample size of participating students, in order to verify the different effect of the scaffoldings on CPS. In addition, future instructional design should carefully consider the educational culture, time constraint, and learning regulation during the idea-centered CPS practices.

## Data Availability

The data that support the findings of this study are available on request from the first author.

## An example of the CPS activity

Please design a set of online learning resources for a geography class in a senior high school. The theme focuses on “regional geography”. The resources can be demonstrated in the form of video, web page, animation, or e-book, etc. The purpose is to help students explore and learn the geographical knowledge of the area. The elements of regional geography include: natural geography (e.g., location, climate, topographic features, hydrology, natural resources, etc.) and cultural geography (e.g., population, city, traffic, industry, agriculture, regional development, etc.). The supporting PPT can be found in the resource section. During the design, the group needs to think about how to trigger students’ learning motivation in online learning contexts and procedures.

## Group A

No scaffolding.

## Group B

In order to complete the design task, you can consider to complete these sub-tasks. First, choose a typical geographical problem in a region (e.g., river management); then, design a learning resource to cover the topic related to the problem; finally, design a specific online learning situation using this online resource to help learners improve learning interest, increase knowledge, and improve performance. You can repeat these sub-tasks to complete the whole design.

## Group C

In order to complete the design task, you can consider to build an instructional design model first (e.g., ADDIE: analysis, design, development, implementation, evaluation), then get familiar with the elements or concepts in this model, and finally design and develop the online learning resources based on this model.

## Part 1: Group collaborative quality

Please score the quality of your group collaboration on the following nine dimensions. Do you agree with the following statement? Please score the quality on a scale of 1–10 with the lowest score of 1 and the highest score of 10.

- Group members maintained a good mutual understanding of the problem to be solved.
- Group members organized the communication well enough to keep continuous discourse with a minimum level of time lost.
- Group members kept a high level of information sharing and collected as much information as possible related to the problem.
- Group members gathered and evaluated alternatives of solutions before reaching a consensus.
- Group members had a clear division of tasks, completed their tasks systematically, moved forward to the solution step by step, and set clear goals or guidance for the sub-tasks.
- Group members had a reasonable time management, properly monitored the remaining time during the collaboration, and assured enough time for the remaining tasks.
- Group members used the technologies smoothly, mastered basic technical skills, and made good use of various functions of the platform.
- Group members interacted with each other in a high quality, respected and encouraged each other to work together, and contributed their own ideas.
- Group members actively contributed to the final group solution and fully used their knowledge and skills during the process.

## Part 2: Student engagement level

- Please score your engagement level on a scale of 1 to 10, with the lowest score of 1 and the highest score of 10.
- What is your contribution in the CPS activities? Please give an example to provide evidence.
- What knowledge worker role did you take in the CPS process, if you have to choose one: a *knowledge learner* (to learn new knowledge), a *knowledge user* (to apply existing knowledge), or a *knowledge builder* (to summarize, synthesize, and build new knowledge). Why? Please give an example to provide evidence.

## Part 3: The usefulness of the scaffolding

- For group A:
  The instructor scaffolded the group collaboration through an online platform with general technical and organizational supports. Did you think the scaffold was useful for the group work? Please score it on a scale of 1–10, with the lowest score of 1 and the highest score of 10, and give detailed reasons of the score.
- For group B:
  The instructor scaffolded the group collaboration through a task-oriented strategy on the platform. Did you think the scaffold was useful for the group work? Please score it on a scale of 1–10 with the lowest score of 1 and the highest score of 10, and give detailed reasons of the score.
- For group C:
  The instructor scaffolded the group collaboration through an idea-oriented strategy on the platform. Did you think the scaffold was useful for the group work? Please score it on a scale of 1–10 with the lowest score of 1 and the highest score of 10, and give detailed reasons of the score.
